# Association of Area-Level Socioeconomic Deprivation With Hypoglycemic and Hyperglycemic Crises in US Adults With Diabetes

**DOI:** 10.1001/jamanetworkopen.2021.43597

**Published:** 2022-01-18

**Authors:** Shaheen Shiraz Kurani, Herbert C. Heien, Lindsey R. Sangaralingham, Jonathan W. Inselman, Nilay D. Shah, Sherita Hill Golden, Rozalina G. McCoy

**Affiliations:** 1 Division of Health Care Delivery Research, Mayo Clinic, Rochester, Minnesota; 2 Mayo Clinic Robert D. and Patricia E. Kern Center for the Science of Health Care Delivery, Rochester, Minnesota; 3 OptumLabs, Eden Prairie, Minnesota; 4 Division of Endocrinology, Diabetes, and Metabolism, Department of Medicine, Johns Hopkins Medicine, Baltimore, Maryland; 5 Office of Diversity, Inclusion, and Health Equity, Johns Hopkins Medicine, Baltimore, Maryland; 6 Division of Community Internal Medicine, Geriatrics, and Palliative Care, Department of Medicine, Mayo Clinic, Rochester, Minnesota

## Abstract

**Importance:**

Social determinants of health play a role in diabetes management and outcomes, including potentially life-threatening complications of severe hypoglycemia and diabetic ketoacidosis (DKA) or hyperglycemic hyperosmolar state (HHS). Although several person-level socioeconomic factors have been associated with these complications, the implications of area-level socioeconomic deprivation are unknown.

**Objective:**

To examine the association between area-level deprivation and the risks of experiencing emergency department visits or hospitalizations for hypoglycemic and hyperglycemic crises (ie, DKA or HHS).

**Design, Setting, and Participants:**

This cohort study used deidentified administrative claims data for privately insured individuals and Medicare Advantage beneficiaries across the US. The analysis included adults with diabetes who met the claims criteria for diabetes between January 1, 2016, and December 31, 2017. Data analyses were performed from November 17, 2020, to November 11, 2021.

**Exposures:**

Area deprivation index (ADI) was derived for each county for 2016 and 2017 using 17 county-level indicators from the American Community Survey. ADI values were applied to patients who were living in each county based on their index dates and were categorized according to county-level ADI quintile (with quintile 1 having the least deprivation and quintile 5 having the most deprivation).

**Main Outcomes and Measures:**

The numbers of emergency department visits or hospitalizations related to the primary diagnoses of hypoglycemia and DKA or HHS (ascertained using validated diagnosis codes in the first or primary position of emergency department or hospital claims) between 2016 and 2019 were calculated for each ADI quintile using negative binomial regression models and adjusted for patient age, sex, health plan type, comorbidities, glucose-lowering medication type, and percentage of White residents in the county.

**Results:**

The study population included 1 116 361 individuals (563 943 women [50.5%]), with a mean (SD) age of 64.9 (13.2) years. Of these patients, 343 726 (30.8%) resided in counties with the least deprivation (quintile 1) and 121 810 (10.9%) lived in counties with the most deprivation (quintile 5). Adjusted rates of severe hypoglycemia increased from 13.54 (95% CI, 12.91-14.17) per 1000 person-years in quintile 1 counties to 19.13 (95% CI, 17.62-20.63) per 1000 person-years in quintile 5 counties, corresponding to an incidence rate ratio of 1.41 (95% CI, 1.29-1.54; *P* < .001). Adjusted rates of DKA or HHS increased from 7.49 (95% CI, 6.96-8.02) per 1000 person-years in quintile 1 counties to 8.37 (95% CI, 7.50-9.23) per 1000 person-years in quintile 5 counties, corresponding to an incidence rate ratio of 1.12 (95% CI, 1.00-1.25; *P* = .049).

**Conclusions and Relevance:**

This study found that living in counties with a high area-level deprivation was associated with an increased risk of severe hypoglycemia and DKA or HHS. The concentration of these preventable events in areas of high deprivation signals the need for interventions that target the structural barriers to optimal diabetes management and health.

## Key Points

**Question:**

Is there an association between county-level deprivation and hypoglycemic and hyperglycemic crises among adults with diabetes?

**Findings:**

In this cohort study of 1 116 361 adults with diabetes, patients who lived in areas of increasing socioeconomic deprivation experienced significantly higher rates of emergency department visits and hospitalizations for severe hypoglycemia and diabetic ketoacidosis or hyperglycemic hyperosmolar state after adjustment for other patient-level risk factors.

**Meaning:**

The findings of this study suggest that county-level socioeconomic deprivation is an independent risk factor for hypoglycemic and hyperglycemic crises, calling for interventions that target the structural barriers to optimal diabetes management and health.

## Teaser

This cohort study examines the geographically based risk factors for emergency department visits and hospitalizations associated with severe hypoglycemia and diabetic ketoacidosis or hyperglycemic hyperosmolar state in insured individuals with diabetes.

## Introduction

Social determinants of health, including individual and area-level factors, play an important role in diabetes incidence, management, and outcomes. In particular, social and economic deprivation may make patients vulnerable to potentially preventable acute complications of severe hypoglycemia and hyperglycemia stemming from inadequate access to health services, medications (insulin and others), healthy food, stable housing, and other facilitators of successful diabetes self-management.^1^ Individual-level factors, such as race and ethnicity,^2^ food insecurity,^3,4,5^ and low income,^6,7,8^ have been associated with an increased risk of hypoglycemia, and much attention has focused on insulin rationing in the setting of rising costs that may lead to severe hyperglycemia.^9^ However, less is known about whether area-level socioeconomic factors contribute to the risk of hypoglycemic and hyperglycemic crises. Identifying and understanding the geographic clustering of poor health outcomes are important for informing the development of population-level medical, public health, and policy interventions that target the underlying structural determinants of health.

One way to quantify geographic disparities is by using the area deprivation index (ADI), a validated composite measure of social determinants of health that spans broad categories of socioeconomic disadvantage for Census-based regions.^10^ ADI is composed of 17 US Census indicators, including domains of poverty, education, housing, and employment,^11^ that reflect general resource availability, educational quality, employment opportunity, and social support,^1^ all of which contribute to the physical, emotional, and financial health of communities and their residents. ADI has been used widely in different geographic areas and health conditions for nearly 2 decades,^12,13,14,15,16^ although it has not been applied to administrative claims data or to acute diabetes complications. It complements individual-level data that may also not be readily available in all settings and provides a comprehensive understanding of entire communities. ADI can be calculated at the Census block group and county levels. Although block group data are more granular, county-level information is more accessible and actionable for public health agencies and policy makers and is therefore used in this study.

Earlier studies conducted among patients with type 1 diabetes found that area-level deprivation was associated with increased risk of diabetic ketoacidosis (DKA)^17^ and all-cause emergency department (ED) visits and hospitalizations.^18^ However, similar studies have not been conducted in the general population of adults with diabetes, more than 90% of whom have type 2 diabetes. Previous studies also have not examined the association between area-level deprivation and acute diabetes complications independent of key patient-level hypoglycemia and hyperglycemia risk factors because such granular clinical data are often scarce in nationwide data sets.

In this study, we addressed these knowledge gaps by examining the association between area-level deprivation and the risks of experiencing ED visits or hospitalizations for hypoglycemic and hyperglycemic crises (ie, DKA or hyperglycemic hyperosmolar state [HHS]). We used long-term person-level data from the OptumLabs Data Warehouse (OLDW; Optum Inc), which allowed us to quantify the associations between ADI and severe hypoglycemia and DKA or HHS while adjusting for known hypoglycemia and hyperglycemia risk factors such as patient age, glucose-lowering treatment regimen, and comorbidity burden.^19,20^ Identifying the area-level factors associated with acute diabetes complications that may be amenable to prevention using optimal diabetes management and care can inform clinical, public health, and policy interventions for reducing disparities and improving health outcomes in at-risk populations.

## Methods

### Study Design and Study Population

This cohort study retrospectively analyzed deidentified medical and pharmacy claims data for commercially insured and Medicare Advantage enrollees from the OLDW, a large nationwide database.^21^ The OLDW contains long-term health information on enrollees, who represent diverse ages, races and ethnicities, and geographic regions across the US. Given that all data were accessed after deidentification,^22^ the study was exempt from review, and the informed consent requirement was waived by the Mayo Clinic Institutional Review Board. We followed the Strengthening the Reporting of Observational Studies in Epidemiology (STROBE) reporting guideline.^23^

We included all individuals who were 18 years or older, had an established diagnosis of diabetes (ascertained using HEDIS [Healthcare Effectiveness Data and Information Set] claims–computable criteria^24^), and had 12 months of baseline enrollment (for baseline covariate ascertainment). Patients entered the cohort between January 1, 2016, and December 31, 2017, and were followed up for outcome ascertainment until either health plan disenrollment or December 31, 2019, whichever occurred first.

### ADI Derivation

County demographic information that was necessary for ADI derivation was ascertained from 5-year estimates of the American Community Survey (ACS),^25^ an annual survey conducted by the US Census Bureau that randomly samples housing units and provides population-level estimates that are representative of the noninstitutionalized population.^25^ We calculated modified ADI scores for all 3142 US counties using 5-year ACS estimates as previously described.^14^ County-level estimates were used because they are the most granular geographic data available for patients in the OLDW and are readily actionable for ultimate end users of these data. Briefly, variables were selected using factor analysis^11,26,27^ and then transformed to a rate per capita for the county. In a modification from the original ADI and consistent with previous studies,^13,14^ the proportions were standardized and then multiplied by the respective weights that were obtained from the factor score coefficient (eTable 1 in the Supplement), and the 17 weighted measures were summed for each county to obtain the base score. ADI values for 2016 were used for patients with index dates in 2016, and ADI values for 2017 were used for patients with index dates in 2017. A US map with county-level ADI data was previously published.^14^

We excluded 240 counties that were not represented in the OLDW data (ie, no patients residing in these counties met the eligibility criteria in the OLDW). As a result, 2902 counties were included in the final analysis. These counties were divided into ADI quintiles for all analyses, with higher ADI values (quintile 5 [Q5]) representing greater area-level deprivation, a method consistent with that in previous research using ADI.^13,14,15,16^

### Primary Outcomes and Independent Variables

We identified the number of ED visits or hospitalizations related to the primary diagnosis of hypoglycemia and DKA or HHS using *International Classification of Diseases, Ninth Revision (ICD-9)* and *International Statistical Classification of Diseases and Related Health Problems, Tenth Revision (ICD-10)* codes (eTable 2 in the Supplement). These numbers were reported per 1000 person-years.

Patient age, sex, and health plan (commercial vs Medicare Advantage) were ascertained from the OLDW enrollment files. The percentage of White residents within each US county was identified from the ACS; we reported on only the White residents for ease of analyses, which is consistent with the method in previous work. White race and ethnicity were self-reported by respondents to the ACS. Individual-level race and ethnicity data were not available in the OLDW along with the county of residence to preserve patient deidentification. Using *ICD-9* and *ICD-10* diagnosis codes, we ascertained comorbidities that were present during the 12 months preceding the index date, including diabetes complications (cardiovascular disease, cerebrovascular disease, peripheral vascular disease, retinopathy, neuropathy, nephropathy, hypoglycemia, and DKA or HHS)^28^ and nonoverlapping Charlson comorbid conditions (mild liver disease, moderate or severe liver disease, dementia, chronic pulmonary disease, nonmetastatic cancer, and metastatic cancer).^29^ Glucose-lowering medications were identified using pharmacy fill data from 120 days before the index date and were classified as shown in eTable 3 in the Supplement. Glucagon fills were ascertained during the 12 months before the index date. These variables were included because of their known association with heightened risks of severe hypoglycemia and/or DKA or HHS.^19,20^

### Statistical Analysis

We calculated overall frequencies (%) and means (SD) for baseline patient characteristics at the time of cohort entry overall and by ADI quintile. Differences across ADI quintiles were assessed using the χ^2^ test of independence. We then ran 2 separate negative binomial regression models to examine the associations between baseline ADI quintiles and severe hypoglycemia and DKA or HHS during the follow-up period. We used Huber-White robust SEs that were clustered at the county level to adjust SEs for county variation. Follow-up time was applied in the negative binomial regression model as exposure time, which by default incorporates the offset in the dispersion formula and is a function of the expected mean [1+α × e^(xβ+offset)^], thus accounting for the varying follow-up time for each patient. Independent variables included in the models were ADI quintile; county-level estimates for percentage of White residents; and patient age, sex, health plan type, glucose-lowering medication type, and comorbidities. Estimated rates of severe hypoglycemia and DKA or HHS were calculated across ADI quintiles for all years using negative binomial regression models combined with a predictive margins statement in Stata, version 15.1 (StataCorp LLC).

In all analyses, 2-sided *P* < .05 was used to indicate statistical significance. Data analyses were conducted using SAS, version 9.4 (SAS Institute Inc) and Stata, version 15.1 (StataCorp LLC) from November 17, 2020, to November 11, 2021.

### Secondary Analyses

First, we examined the association between ADI and severe hypoglycemia and DKA or HHS and then calculated the estimated rates of severe hypoglycemia and DKA or HHS, adjusting for only patient demographic characteristics (age, sex, and health plan) and the percentage of White residents in their county. Second, the associations between ADI and severe hypoglycemia and DKA or HHS were examined separately for age subgroups (18-44, 45-64, 65-74, or ≥75 years), by patient sex (female or male), by health plan (commercial or Medicare Advantage), and by type of treatment regimen (bolus insulin with or without basal insulin or noninsulin drugs, basal insulin with or without noninsulin drugs, or noninsulin drugs only).

## Results

The study population comprised 1 116 361 adults with diabetes who resided across 2902 counties in the US. These individuals had a mean (SD) age of 64.9 (13.2) years and included 563 943 women (50.5%) and 552 418 men (49.5%), of whom 419 194 (37.6%) had commercial health insurance and 697 167 (62.5%) were Medicare Advantage beneficiaries. The mean (SD) duration of observation was 854.6 (446) days. Overall, 343 726 patients (30.8%) lived in the quintile of counties with the least deprivation (Q1), whereas 121 810 patients (10.9%) lived in the quintile of counties with the most deprivation (Q5) (Table 1). Patients who resided in counties with more deprivation (ie, higher ADI quintiles) were progressively older, more likely to be women, more likely to have Medicare Advantage vs commercial health insurance, and more likely to have a higher prevalence of all examined comorbidities and diabetes complications. Counties with more deprivation also had a lower percentage of White residents (mean [SD], Q5: 65.8% [22.2%] vs Q1: 75.6% [15.9%]).

**Table 1.** Characteristics of Study Population

Characteristic	ADI quintile, No. (%)^a^					*P* value
	Q1	Q2	Q3	Q4	Q5	
No. of patients	343 726 (30.8)	265 190 (23.8)	218 133 (19.5)	167 502 (15.0)	121 810 (10.9)	
Age, mean (SD), y	64.09 (13.8)	64.79 (13.3)	64.46 (13.2)	65.16 (12.8)	67.43 (11.7)	<.001
Age category, y						<.001
18-44	31 267 (9.1)	21 107 (8.0)	17 260 (7.9)	11 710 (7.0)	5548 (4.6)	
45-64	128 128 (37.3)	95 272 (35.9)	82 312 (37.7)	60 012 (35.8)	35 587 (29.2)	
65-74	102 625 (29.9)	83 761 (31.6)	67 375 (30.9)	54 568 (32.6)	46 103 (37.9)	
≥75	81 706 (23.8)	65 050 (24.5)	51 186 (23.5)	41 212 (24.6)	34 572 (28.4)	
Sex						<.001
Female	165 224 (48.1)	131 329 (49.5)	112 021 (51.4)	87 280 (52.1)	68 089 (55.9)	
Male	178 502 (51.9)	133 861 (50.5)	106 112 (48.7)	80 222 (47.9)	53 721 (44.1)	
Health plan						<.001
Commercial	152 267 (44.3)	102 944 (38.8)	84 058 (38.5)	56 629 (33.8)	23 296 (19.1)	
Medicare Advantage	191 459 (55.7)	162 246 (61.2)	134 075 (61.5)	110 873 (66.2)	98 514 (80.9)	
% White residents in county, mean (SD)^b^	75.63 (15.9)	74.69 (15.8)	72.45 (14.5)	73.02 (16.1)	65.79 (22.2)	<.001
Glucose-lowering medications						
No pharmacy fills	89 096 (25.9)	70 681 (26.7)	57 043 (26.2)	42 852 (25.6)	29 796 (24.5)	<.001
Insulin						
Basal human	5682 (1.7)	4952 (1.9)	4496 (2.1)	4085 (2.4)	3419 (2.8)	<.001
Basal analog	57 837 (16.8)	43 372 (16.4)	36 992 (17.0)	28 749 (17.2)	21 487 (17.6)	<.001
Bolus human	5192 (1.5)	4784 (1.8)	4491 (2.1)	4255 (2.5)	3574 (2.9)	<.001
Bolus analog	41 105 (12.0)	29 357 (11.1)	23 845 (10.9)	18 413 (11.0)	12 315 (10.1)	<.001
DPP-4 inhibitors	37 959 (11.0)	28 483 (10.7)	24 523 (11.2)	19 321 (11.5)	15 311 (12.6)	<.001
GLP-1 receptor agonists	17 595 (5.1)	12 676 (4.8)	11 624 (5.3)	8395 (5.0)	5231 (4.3)	<.001
Metformin hydrochloride	172 428 (50.2)	130 337 (49.2)	106 403 (48.8)	81 243 (48.5)	59 368 (48.7)	<.001
SGLT2 inhibitors	17 807 (5.2)	13 650 (5.2)	12 468 (5.7)	8703 (5.2)	5264 (4.3)	<.001
Sulfonylurea	82 159 (23.9)	65 718 (24.8)	53 645 (24.6)	43 801 (26.2)	32 899 (27.0)	<.001
Thiazolidinediones	15 107 (4.4)	12 057 (4.6)	10 328 (4.7)	7809 (4.7)	5436 (4.5)	<.001
Other medications^c^	931 (0.3)	687 (0.3)	578 (0.3)	380 (0.2)	352 (0.3)	.01
Glucagon	4196 (1.2)	2450 (0.9)	1762 (0.8)	1132 (0.7)	545 (0.5)	<.001
Diabetes complications and comorbidities						
Cancer	30 573 (8.9)	24 138 (9.1)	18 958 (8.7)	14 304 (8.5)	11 000 (9.0)	<.001
Cardiovascular disease	112 665 (32.8)	92 124 (34.7)	78 062 (35.8)	58 605 (35.0)	50 274 (41.3)	<.001
Cerebrovascular disease	30 867 (9.0)	25 468 (9.6)	21 949 (10.1)	16 456 (9.8)	13 459 (11.1)	<.001
Chronic pulmonary disease	64 842 (18.9)	55 531 (20.9)	48 869 (22.4)	38 747 (23.1)	30 897 (25.4)	<.001
Dementia	17 779 (5.2)	12 480 (4.7)	10 793 (5.0)	7933 (4.7)	7472 (6.1)	<.001
DKA or HHS	1868 (0.5)	1490 (0.6)	1376 (0.6)	988 (0.6)	672 (0.6)	<.001
Metastatic cancer	4417 (1.3)	3496 (1.3)	2833 (1.3)	2180 (1.3)	1602 (1.3)	.83
Mild liver disease	23 712 (6.9)	19 204 (7.2)	15 854 (7.3)	11 169 (6.7)	8279 (6.8)	<.001
Moderate or severe liver disease	2016 (0.6)	1625 (0.6)	1542 (0.7)	1154 (0.7)	937 (0.8)	<.001
Peripheral vascular disease	51 784 (15.1)	40 588 (15.3)	37 100 (17.0)	25 488 (15.2)	25 070 (20.6)	<.001
Nephropathy	73 695 (21.4)	58 384 (22.0)	51 500 (23.6)	37 696 (22.5)	32 788 (26.9)	<.001
Neuropathy	82 905 (24.1)	68 998 (26.0)	61 293 (28.1)	45 864 (27.4)	40 694 (33.4)	<.001
Retinopathy	51 947 (15.1)	38 720 (14.6)	33 184 (15.2)	21 987 (13.1)	20 833 (17.1)	<.001
Severe hypoglycemia	2929 (0.8)	2345 (0.9)	2373 (1.1)	1746 (1.0)	1753 (1.4)	<.001

Abbreviations: ADI, area deprivation index; DKA, diabetic ketoacidosis; DPP-4, dipeptidyl-peptidase 4; GLP-1, glucagon-like peptide-1; HHS, hyperglycemic hyperosmolar state; SGLT2, sodium-glucose cotransporter 2.

^a^ Baseline characteristics of patients included in the study were stratified by ADI quintile of the county of residence from least deprivation (Q1) to most deprivation (Q5). Results are shown as number (%) unless specified otherwise.

^b^ Only the White residents per county were included for ease of analyses, which is consistent with the method in previous work. White race and ethnicity were self-reported by respondents to the American Community Survey.^25^

^c^ Other glucose-lowering medications included glinides, amylin analogs, and α-glucosidase inhibitors.

There were significant differences in the distribution of glucose-lowering medications used by patients at different levels of deprivation (Table 1). Overall, 24.5% of patients living in Q5 counties had no fills for glucose-lowering medications during the 120 days preceding the index date compared with 25.9% of patients in Q1 counties. Patients living in Q5 compared with Q1 counties were more likely to be treated with human basal insulin (2.8% vs 1.7%; *P* < .001), human bolus insulin (2.9% vs 1.5%; *P* < .001), sulfonylurea (27.0% vs 23.9%; *P* < .001), and dipeptidyl-peptidase 4 inhibitor (12.6% vs 11.0%; *P* < .001). Analog basal insulin was also used more frequently by patients in Q5 than Q1 (17.6% vs 16.8%; *P* < .001), but analog bolus insulin was used less often (10.1% vs 12.0%; *P* < .001). Metformin hydrochloride (48.7% vs 50.2%; *P* < .001), sodium-glucose cotransporter 2 inhibitors (4.3% vs 5.2%; *P* < .001), and glucagon-like peptide-1 receptor agonists (4.3% vs 5.1%; *P* < .001) were used less often by patients in Q5 than Q1. Glucagon was rarely filled overall but even less so by people residing in Q5 compared with Q1 counties (0.5% vs 1.2%; *P* < .001).

### Area-Level Deprivation, Severe Hypoglycemia, and DKA or HHS

Adjusted rates of severe hypoglycemia increased from 13.54 (95% CI, 12.91-14.17) per 1000 person-years in Q1 counties to 19.13 (95% CI, 17.62-20.63) per 1000 person-years in Q5 counties (Figure). This result corresponded to an incidence rate ratio (IRR) of 1.41 (95% CI, 1.29-1.54; *P* < .001) for Q5 vs Q1 (Table 2). Adjusted rates of DKA or HHS increased from 7.49 (95% CI, 6.96-8.02) per 1000 person-years in Q1 counties to 8.37 (95% CI, 7.50-9.23) per 1000 person-years in Q5 counties. This finding corresponded to an IRR of 1.12 (95% CI, 1.00-1.25; *P* = .049) for Q5 vs Q1. For each percentage point increase in the White population of each county, the risk decreased by 33% (IRR, 0.67; 95% CI, 0.62-0.72; *P* < .001) for severe hypoglycemia and by 20% (IRR, 0.80; 95% CI, 0.67-0.95; *P* = .01) for DKA or HHS.

**Figure.**
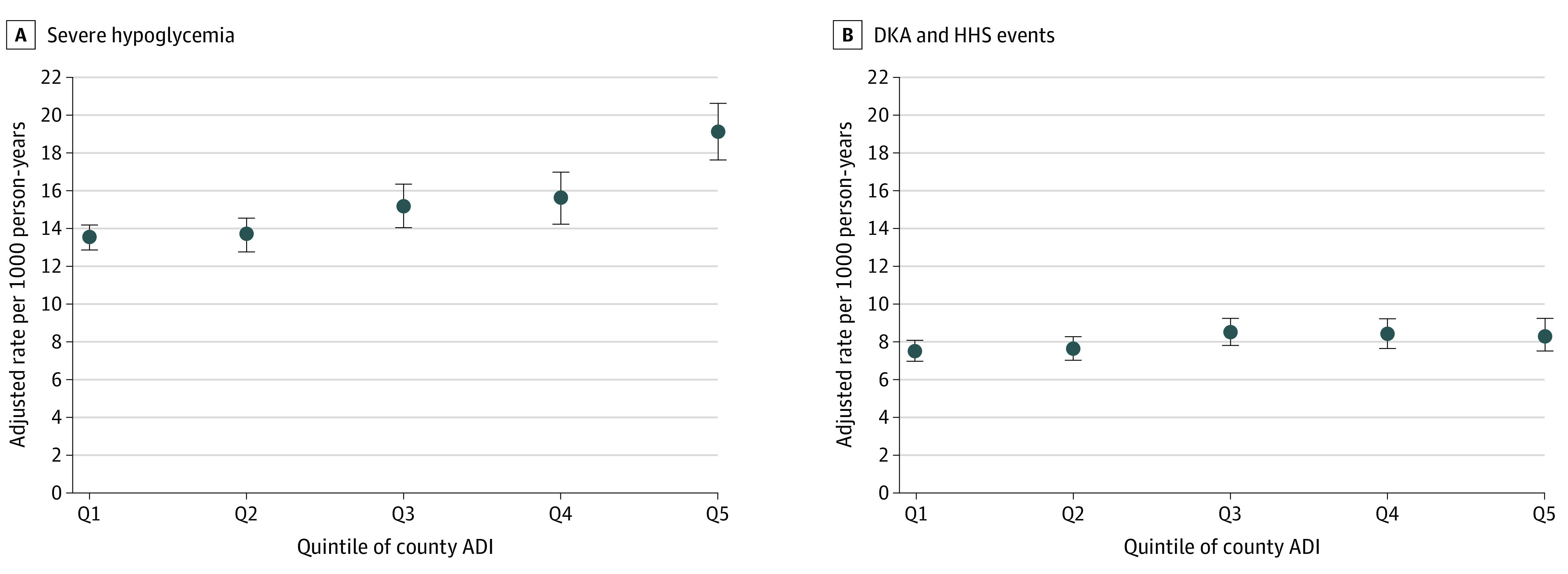
Adjusted Rates of Severe Hypoglycemic and Diabetic Ketoacidosis (DKA) or Hyperglycemic Hyperosmolar State (HHS) Events, Stratified by Area Deprivation Index (ADI) Quintile of the County of Residence Rates were adjusted for individual patient age, sex, health plan type, glucose-lowering medications, and comorbidities as well as county-level estimates for percentage of White residents.

**Table 2.** Risk Factors of Severe Hypoglycemia and Diabetic Ketoacidosis or Hyperglycemic Hyperosmolar State^a^

Variable	Hypoglycemia		DKA or HHS	
	IRR (95% CI)	*P* value	IRR (95% CI)	*P* value
% White residents in county	0.67 (0.62-0.72)	<.001	0.80 (0.67-0.95)	.01
ADI quintile				
Q1 (least deprivation)	1 [Reference]		1 [Reference]	
Q2	1.01 (0.94-1.09)	.84	1.02 (0.94-1.11)	.61
Q3	1.12 (1.03-1.22)	.009	1.14 (1.04-1.24)	.003
Q4	1.15 (1.05-1.27)	.004	1.12 (1.02-1.24)	.02
Q5 (most deprivation)	1.41 (1.29-1.54)	<.001	1.12 (1.00-1.25)	.049
Sex				
Female	1 [Reference]		1 [Reference]	
Male	0.93 (0.90-0.96)	<.001	0.92 (0.88-0.97)	.002
Health plan				
Commercial	1 [Reference]		1 [Reference]	
Medicare Advantage	2.40 (2.24-2.57)	<.001	1.82 (1.68-1.98)	<.001
Age category, y				
18-44	1 [Reference]		1 [Reference]	
45-64	0.79 (0.72-0.86)	<.001	0.35 (0.32-0.38)	<.001
65-74	0.66 (0.60-0.73)	<.001	0.15 (0.13-0.16)	<.001
≥75	0.83 (0.76-0.92)	<.001	0.12 (0.11-0.13)	<.001
Glucose-lowering medications				
No pharmacy fills	0.68 (0.64-0.72)	<.001	0.77 (0.70-0.84)	<.001
Insulin				
Basal human	2.27 (2.04-2.53)	<.001	1.45 (1.23-1.72)	<.001
Basal analog	2.00 (1.92-2.08)	<.001	1.77 (1.66-1.88)	<.001
Bolus human	1.81 (1.63-2.01)	<.001	1.86 (1.58-2.20)	<.001
Bolus analog	2.36 (2.26-2.46)	<.001	2.53 (2.35-2.73)	<.001
DPP-4 inhibitors	0.84 (0.79-0.88)	<.001	0.84 (0.77-0.92)	<.001
GLP-1 receptor agonists	0.66 (0.61-0.71)	<.001	0.71 (0.63-0.79)	<.001
Metformin	0.57 (0.55-0.59)	<.001	0.55 (0.51-0.58)	<.001
SGLT2 inhibitors	0.70 (0.63-0.77)	<.001	1.25 (1.12-1.40)	<.001
Sulfonylurea	1.68 (1.61-1.75)	<.001	0.92 (0.86-0.99)	.02
Thiazolidinediones	1.02 (0.95-1.09)	.58	0.94 (0.82-1.08)	.36
Other medications^b^	1.08 (0.87-1.34)	.49	0.95 (0.57-1.61)	.86
Glucagon	1.87 (1.66-2.10)	<.001	1.75 (1.53-2.00)	<.001
Diabetes complications and other comorbidities				
Cardiovascular disease	1.21 (1.17-1.25)	<.001	0.93 (0.87-0.98)	.01
Cancer	1.00 (0.95-1.05)	.91	0.89 (0.80-0.98)	.02
Cerebrovascular disease	1.19 (1.14-1.25)	<.001	1.12 (1.03-1.21)	.007
Chronic pulmonary disease	1.12 (1.08-1.16)	<.001	0.95 (0.89-1.01)	.08
Dementia	1.10 (1.02-1.18)	.01	1.21 (1.08-1.36)	.002
DKA or HHS	3.21 (2.80-3.69)	<.001	22.48 (20.23-24.97)	<.001
Metastatic cancer	1.34 (1.18-1.51)	<.001	1.23 (0.98-1.55)	.08
Mild liver disease	0.99 (0.93-1.06)	.87	1.07 (0.97-1.18)	.20
Moderate or severe liver disease	1.70 (1.45-1.99)	<.001	1.17 (0.92-1.50)	.20
Nephropathy	1.53 (1.47-1.58)	<.001	1.22 (1.15-1.29)	<.001
Neuropathy	1.35 (1.31-1.40)	<.001	1.41 (1.34-1.49)	<.001
Peripheral vascular disease	1.15 (1.11-1.20)	<.001	1.03 (0.96-1.11)	.42
Retinopathy	1.40 (1.35-1.45)	<.001	1.22 (1.14-1.30)	<.001
Severe hypoglycemia	7.57 (7.02-8.16)	<.001	4.04 (3.58-4.57)	<.001

Abbreviations: ADI, area deprivation index; DKA, diabetic ketoacidosis; DPP-4, dipeptidyl-peptidase 4; GLP-1, glucagon-like peptide-1; HHS, hyperglycemic hyperosmolar state; IRR, incidence rate ratio; SGLT2, sodium-glucose cotransporter 2.

^a^ Results of negative binomial regression models were adjusted for all variables shown.

^b^ Other glucose-lowering medications included glinides, amylin analogs, and α-glucosidase inhibitors.

Results were similar in partially adjusted models, which examined the association between ADI and severe hypoglycemia and DKA or HHS, adjusting only for patient demographic characteristics (age, sex, and health plan) and the percentage of the White population in the county. These data are detailed in eTables 4 and 5 in the Supplement.

### Individual-Level Risk Factors for Severe Hypoglycemia and DKA or HHS

Men were less likely than women to experience both severe hypoglycemia (IRR, 0.93; 95% CI, 0.90-0.96; *P* < .001) and DKA or HHS (IRR, 0.92; 95% CI, 0.88-0.97; *P* = .002) (Table 2). The risk of experiencing both severe hypoglycemia (aged ≥75 years: IRR, 0.83: 95% CI, 0.76-0.92; *P* < .001) and DKA or HHS (aged ≥75 years: IRR, 0.12; 95% CI, 0.11-0.13; *P* < .001) was lowest among older adults vs those 18 to 44 years of age. Patients who were treated with sulfonylureas and insulin were more likely to experience severe hypoglycemia, whereas patients who were treated with sodium-glucose cotransporter 2 inhibitors and those requiring insulin therapy were more likely to experience DKA or HHS.

The strongest risk factors for both events were previous such events. For severe hypoglycemia, previous severe hypoglycemia increased the risk of recurrent events by an IRR of 7.57 (95% CI, 7.02-8.16; *P* < .001), and DKA or HHS increased the risk by an IRR of 3.21 (95% CI, 2.80-3.69; *P* < .001). For DKA or HHS, previous DKA or HHS increased the risk of recurrent events by an IRR of 22.48 (95% CI, 20.23-24.97; *P* < .001) and severe hypoglycemia increased it by an IRR of 4.04 (95% CI, 3.58-4.57; *P* < .001).

### Subgroup Analyses

The estimated rates of severe hypoglycemia and DKA or HHS as a function of ADI for subgroups of patient age, sex, health plan, and glucose-lowering treatment regimen are presented in eTables 6-9 in the Supplement. The rates of severe hypoglycemia were higher in patients in all age groups who were living in counties with more deprivation. In contrast, for DKA or HHS, the association with ADI was significant only among patients aged 18 to 44 years living in Q5 vs Q1 counties (IRR, 1.42; 95% CI, 1.11-1.82; *P* = .006). The association between ADI and severe hypoglycemia was significant among both men (IRR, 1.32; 95% CI, 1.19-1.47; *P* < .001) and women (IRR, 1.48; 95% CI, 1.34-1.63; *P* < .001) living in Q5 vs Q1 counties, whereas the association with DKA or HHS was significant only among women (IRR, 1.17; 95% CI, 1.02-1.35; *P* = .03) but not men (IRR, 1.06; 95% CI, 0.91-1.23; *P* = .46) living in Q5 vs Q1 counties.

Rates of severe hypoglycemia increased with higher ADI regardless of the patient’s diabetes treatment regimen (bolus insulin–treated patients living in Q5 vs Q1 counties: IRR, 1.30 [95% CI, 1.18-1.43; *P* < .001]; basal insulin–treated patients living in Q5 vs Q1 counties: IRR, 1.33 [95% CI, 1.18-1.50; *P* < .001]; and noninsulin-treated patients living in Q5 vs Q1 counties: IRR, 1.48 [95% CI, 1.32-1.65; *P* < .001]). In contrast, for DKA or HHS, the association with ADI quintile did not reach statistical significance for any of the treatment regimens. In the commercial insurance group, rates of both severe hypoglycemia (patients living in Q5 vs Q1 counties: IRR, 1.31; 95% CI, 1.10-1.56; *P* = .003) and DKA or HHS (patients in Q5 vs Q1 counties: IRR, 1.25; 95% CI, 1.05-1.50; *P* = .01) increased with greater deprivation. In contrast, the Medicare Advantage beneficiaries experienced increasing rates of severe hypoglycemia (patients in Q5 vs Q1 counties: IRR, 1.43; 95% CI, 1.30-1.57; *P* < .001), but there was no difference in the rates of DKA or HHS (patients in Q5 vs Q1 counties: IRR, 1.07; 95% CI, 0.93-1.22; *P* = .34).

## Discussion

Social determinants of health, including person-level and area-level factors, are associated with the risk of experiencing dangerous, yet potentially preventable, hypoglycemic and hyperglycemic crises. This study found an independent and consistent association between area-level deprivation and hospitalizations for both severe hypoglycemia and DKA or HHS. After adjusting for pertinent individual- and treatment-level risk factors, we found that patients living in the quintile of counties with the most deprivation had a 41% higher risk of severe hypoglycemia and a 12% higher risk of DKA or HHS compared with people living in the quintile of counties with the least deprivation.

We believe this work builds on a body of evidence revealing pervasive racial, ethnic, and socioeconomic disparities in diabetes management and health outcomes by identifying a geographic concentration of potentially preventable acute complications of diabetes. The geographic concentration of these ED visits and hospitalizations in areas of high deprivation provides actionable information to health systems, public health agencies, and governments and signals the need for targeted interventions and potential use of ADI as a population-level marker of vulnerability to poor health outcomes.

There are several potential explanations for the high rates of hypoglycemic and hyperglycemic crises in areas of high deprivation. Socioeconomically deprived counties may have fewer resources available to support high-quality diabetes care, including availability of endocrinologists, primary care physicians with expertise in diabetes management, and certified diabetes education and care specialists.^5^ There may be fewer options for obtaining adequate nutritious food and engaging in physical activity.^5^ Structural racism may underlie many of these geographic inequities,^30^ resulting from deeply rooted discriminatory housing policies^31,32^ that concentrated racial and ethnic minority individuals into neighborhoods with reduced access to education, employment, healthy food outlets, and safe spaces for physical activity as well as higher levels of pollution and environmental stress.^31,33,34,35,36,37,38,39,40,41^ Past research has linked the rates of obesity to county-level indicators of structural racism, including racial inequality in homeownership, unemployment, poverty, and school racial segregation.^42,43,44^ These same geographically defined factors are likely associated with the risks of hypoglycemia and hyperglycemia and suggest potential interventions targeted at alleviating structural barriers to optimal diabetes management and health.

Residential racial segregation is one of the strongest factors in the disparities among Black individuals.^38,39,45^ Because ADI does not include explicit measures of race or ethnicity, we were able to concurrently examine the association of neighborhood racial composition with the risks of severe hypoglycemia and DKA or HHS. For each percentage point increase in the White population of each county, the risk of severe hypoglycemia decreased by 33% and the risk of DKA or HHS decreased by 20%, independent of the county ADI. Although this data set lacked individual-level race information, this finding is consistent with that in previous work that demonstrated higher rates of severe hypoglycemia^20^ and DKA or HHS^19^ in Black patients compared with White patients with diabetes. Black patients are more likely to be treated by clinicians^46^ and health systems^47^ that deliver lower-quality services and/or have fewer resources to optimally care for their patients,^46^ which can lead to worse health outcomes. Black neighborhoods have fewer primary care physicians and specialists, decreasing the residents’ access to high-quality medical care.^45^ As a result of structural racism and geographic segregation, Black patients are also more likely to reside in less walkable neighborhoods^48^ with fewer healthy food options^33,49,50^ and limited access to high-quality diabetes care, similarly worsening glycemic control. Thus, socioeconomically deprived neighborhoods of racial and ethnic minority individuals need intensive, multifaceted interventions to improve diabetes management and overall health.^5^

We believe this work adds to the robust literature that demonstrates an association between low-income and other person-level socioeconomic barriers and poor glycemic control.^5^ A previous work found that low-income patients were significantly more likely to experience severe hypoglycemia^20^ and DKA or HHS.^19^ ADI provides a composite estimation of poverty, education, housing, and employment within a geographic area,^11^ therefore encompassing the characteristics of both the people who live in this area and the area itself. The association between deprivation, both individual and community levels, and hypoglycemic and hyperglycemic crises likely has many factors, including food insecurity,^3,4,6^ rationing of medications^9^ and/or food,^51,52^ less predictable and more physically demanding employment, and limited health literacy.^53^ Although this study cannot identify the independent individual and contextual factors in the risks of severe hypoglycemia and DKA or HHS or ascertain exactly which aspects of area-level deprivation are involved in these events, we aimed to assess whether broad socioeconomic geographic constructs like ADI could be used to identify geographic clusters of susceptibility to the acute complications of diabetes. Because individual-level information about income is often not available at the point of care, ADI provides an efficient framework for using area-level socioeconomic information to inform and guide resource allocation, identification of at-risk individuals, and care delivery that incorporates appropriate wraparound services.

The subgroup analyses in the present study demonstrated a consistent association between greater area-level deprivation and higher rates of severe hypoglycemic events. However, the association between ADI and DKA or HHS varied across the different age groups. Specifically, the rate of DKA or HHS increased with higher ADI in younger patients (18-44 years) but not in older patients, whereas the association between ADI and severe hypoglycemia was significant in all age groups. Because DKA and HHS are more likely to occur when patients do not use sufficient glucose-lowering medications, specifically insulin, this association suggests cost-related rationing, nonadherence, or inability to obtain the medications that patients need to adequately manage their diabetes. This heterogeneity underscores the importance of social determinants of health in younger patients, among whom there may be a greater prevalence of financial instability and underinsurance^51,54^ and thus difficulty with paying for their insulin. They also need to balance the demands of their diabetes with other changing life needs, such as employment, education, and family. The findings of this study reinforce that younger patients may benefit from additional diabetes management support, especially if they reside in communities with a high ADI. Although this study did not include diabetes type, it is likely that younger patients have type 1 diabetes and thus are most susceptible to DKA or HHS.

The urgency of addressing the geographic concentrations of poor health outcomes among people with diabetes was underscored by the 2020 American Diabetes Association report on social determinants of health and diabetes,^5^ which focused on 5 domains of social determinants of health, all of which are either explicitly or implicitly geographic: socioeconomic status, neighborhood and physical environment, food environment, health care, and social context. This report^5^ and other recent publications^55^ also provide a framework for tangible policy interventions to address the structural and systemic factors in poor health outcomes. First, we must recognize the presence of geographically concentrated inequities in health care access, quality, and outcomes and understand the structural factors associated with these disparities. Second, policies must target the root causes of health care inequities by districting for and funding affordable housing, safe outdoor spaces, gyms, grocery stores, and clinical facilities as well as by investing in childcare, education, and well-paying jobs. Third, medical and scientific communities must engage, support, and retain professionals from and within the communities they serve. Fourth, health systems should partner with communities and community leaders to deliver comprehensive, patient-centered care both within and outside of clinic walls. Several neighborhood-level interventions have demonstrated improved health outcomes, particularly with respect to obesity, nutrition, and physical activity.^5^ For glycemic control specifically, the Moving to Opportunity for Fair Housing demonstration project exemplifies how moving from high-poverty to low-poverty Census tracts results in better glycemic control and decreased rates of obesity.^56^ It is important to consider and learn from such interventions to improve diabetes management and ultimately prevent acute and chronic diabetes complications.^57^

To our knowledge, this national study was the first to examine the association between area-level deprivation and severe hypoglycemia and DKA or HHS, building on earlier work that was limited to small geographic areas^17,58^ or people with type 1 diabetes.^17,18,59^ The findings of this study provide a framework for identifying area-level factors and populations that are disproportionately at risk for hypoglycemic and hyperglycemic crises. The neighborhoods in which people live inform the structural conditions that ultimately shape their health, independent of their individual-level characteristics. By simultaneously examining both area-level and individual-level risk factors for severe dysglycemia, this study highlighted the health implications of living in areas of concentrated poverty and deprivation. Thus, to identify and reverse the mechanisms of this association, we need to directly engage with people living with diabetes amid the realities of their built environments. Qualitative and mixed-methods studies that use purposive samples of residents from contrasting neighborhoods can elicit the potential implications of available clinical, social, and financial support services; community engagement and use of community services; access to sufficient and nutritious food; neighborhood safety and walkability; and culture of health for health behaviors and outcomes in diabetes. These findings can, in turn, pave the way toward targeted interventions for reducing health disparities and improving the health and well-being of people living with diabetes.

### Limitations

This study has several limitations. We considered only hypoglycemic and DKA or HHS events that culminated in an ED visit or hospital stay. This approach tends to miss most of the severe hypoglycemic events^53,60,61^ and an unknown amount of severe hyperglycemic events. The need for ED or hospital care to manage hypoglycemia and DKA or HHS may be affected not only by event severity but also by individual- and area-level factors. Older patients and patients with multiple or advanced comorbidities, with food^4,62^ or housing insecurity,^63^ without adequate diabetes self-management education, without access to ambulatory care, and without caregiver or other sources of support are all more likely to be brought to the hospital. At the same time, low-income patients may seek to avoid the high costs of ED care and even refuse ED transport if they are treated for hypoglycemia by emergency medical services. We did not have data on diabetes type; however, our goal was to evaluate population-level geographic risk factors of hypoglycemic and hyperglycemic crises that would be agnostic of information that is not readily available on such a scale. Furthermore, we did not have access to individual-level race and ethnicity or income information for patients who were included in the study because these data were not available with county-level geographic location to ensure patient deidentification within the OLDW. The 1.1 million patients who were included in the study were also not representative of the overall US population, as they all had some health insurance coverage (commercial, including high-deductible, plans or Medicare Advantage). Nevertheless, we believe the findings are informative for patients with health insurance given that they may be assumed to be less susceptible to the adverse consequences of area-level deprivation compared with patients without health insurance or with Medicaid coverage.

## Conclusions

This cohort study found an association between area-level deprivation and severe hypoglycemia and DKA or HHS. Patients who lived in US counties that had the highest socioeconomic deprivation had a 41% higher risk of severe hypoglycemia and a 12% higher risk of DKA or HHS compared with those who lived in counties with the least deprivation. The geographic concentration of preventable ED visits and hospitalizations in areas of high deprivation signals the need for interventions that target the structural barriers to optimal diabetes management and health.

## Notes

Supplement. **eTable 1.** American Community Survey Census Indicators **eTable 2.** Codes Used to Ascertain Hypoglycemia and DKA/HHS-Related Hospitalizations and Emergency Department Visits **eTable 3.** Classification of Glucose-Lowering Medications **eTable 4.** Risk Factors of Severe Hypoglycemia and DKA/HHS (Sensitivity Analysis Using the Partially Adjusted Model) **eTable 5.** Estimated Rates of Severe Hypoglycemia and DKA/HHS (Sensitivity Analysis Using the Partially Adjusted Model) **eTable 6.** Estimated Rates of Severe Hypoglycemia and DKA/HHS in Subgroups of Patient Age **eTable 7.** Estimated Rates of Severe Hypoglycemia and DKA/HHS in Subgroups of Patient Sex **eTable 8.** Estimated Rates of Severe Hypoglycemia and DKA/HHS in Subgroups of Glucose-Lowering Therapy **eTable 9.** Estimated Rates of Severe Hypoglycemia and DKA/HHS in Subgroups of Patient Health Plans
eReference
