# Supplementary material for: Association of Area-Level Socioeconomic Deprivation With Hypoglycemic and Hyperglycemic Crises in US Adults With Diabetes
Source: JAMA Netw Open. 2022 Jan 18;5(1):e2143597. doi: 10.1001/jamanetworkopen.2021.43597 (PMC8767428; doi:10.1001/jamanetworkopen.2021.43597)
Supplement: Supplement. — eTable 1. American Community Survey Census Indicators eTable 2. Codes Used to Ascertain Hypoglycemia and DKA/HHS-Related Hospitalizations and Emergency Department Visits eTable 3. Classification of Glucose-Lowering Medications eTable 4. Risk Factors of Severe Hypoglycemia and DKA/HHS (Sensitivity Analysis Using the Partially Adjusted Model) eTable 5. Estimated Rates of Severe Hypoglycemia and DKA/HHS (Sensitivity Analysis Using the Partially Adjusted Model) eTable 6. Estimated Rates of Severe Hypoglycemia and DKA/HHS in Subgroups of Patient Age eTable 7. Estimated Rates of Severe Hypoglycemia and DKA/HHS in Subgroups of Patient Sex eTable 8. Estimated Rates of Severe Hypoglycemia and DKA/HHS in Subgroups of Glucose-Lowering Therapy eTable 9. Estimated Rates of Severe Hypoglycemia and DKA/HHS in Subgroups of Patient Health Plans eReference [file jamanetwopen-e2143597-s001.pdf]

Supplementary Online Content

Kurani SS, Heien HC, Sangaralingham LR, et al. Association of area-level socioeconomic deprivation with hypoglycemic and hyperglycemic crises in US adults with diabetes. *JAMA Netw Open*. 2022;5(1):e2143597. doi:10.1001/jamanetworkopen.2021.43597

eTable 1. American Community Survey Census Indicators
eTable 2. Codes Used to Ascertain Hypoglycemia and DKA/HHS-Related Hospitalizations and Emergency Department Visits
eTable 3. Classification of Glucose-Lowering Medications
eTable 4. Risk Factors of Severe Hypoglycemia and DKA/HHS (Sensitivity Analysis Using the Partially Adjusted Model)
eTable 5. Estimated Rates of Severe Hypoglycemia and DKA/HHS (Sensitivity Analysis Using the Partially Adjusted Model)
eTable 6. Estimated Rates of Severe Hypoglycemia and DKA/HHS in Subgroups of Patient Age
eTable 7. Estimated Rates of Severe Hypoglycemia and DKA/HHS in Subgroups of Patient Sex
eTable 8. Estimated Rates of Severe Hypoglycemia and DKA/HHS in Subgroups of Glucose-Lowering Therapy
eTable 9. Estimated Rates of Severe Hypoglycemia and DKA/HHS in Subgroups of Patient Health Plans
eReference

This supplementary material has been provided by the authors to give readers additional information about their work.

© 2022 Kurani SS et al. *JAMA Network Open.*

eTable 1. American Community Survey Census Indicators

Shown are the survey census indicators, table references, and factor score coefficients from 2012-2017 that were used for area deprivation index (ADI) derivation.

U.S. Census Indicator	2012-2017 ACS Table Reference, 5-yr estimates	Factor Score Coefficients	
		**2016**	**2017**
Median family income	B19013	-0.16087	-0.16993
Income disparity	B19001	0.08019	0.06799
Families below poverty level	B17010	0.12555	0.12298
Percent population below 150% poverty threshold	C17002	0.22914	0.23659
Single parent household with dependents <18 years old	B23008	0.03817	0.04165
Households without a motor vehicle	B25044	0.05666	0.05646
Households without a telephone	B25043	0.00685	0.00892
Occupied housing units without complete plumbing	B25016	0.02692	0.02963
Owner occupied housing units	B25003	-0.00888	-0.00733
Households with >1 person per room	B25014	0.03546	0.03747
Median monthly mortgage	B25088	-0.13578	-0.13004
Median gross rent	B25064	-0.05922	-0.06295
Median home value	B25077	-0.07345	-0.0749
Employed persons ≥16 years old in white collar occupation	C24010	-0.02079	-0.01947
Civilian labor force unemployed (aged ≥16)	B23025	0.0228	0.02451
Population aged ≥25 years with <9-year education	B15003	0.00431	0.01132
Population aged ≥25 years with at least a high school education	B15003	-0.22112	-0.21015

© 2022 Kurani SS et al. *JAMA Network Open.*

eTable 2. Codes Used to Ascertain Hypoglycemia and DKA/HHS-Related Hospitalizations and Emergency Department Visits

Health condition	ICD-9 codes	ICD-10 codes
**Hypoglycemia**	251.0, 251.1, 251.2, 270.3, 962.3 or 250.8x (and not 259.8, 272.7, 681.xx, 682.xx, 686.9x, 707.1-707.9, 709.3, 730.0-730.2, 731.8) *per Ginde, et al(1)*	E08.641, E08.649, E09.641, E09.649, E10.641, E10.649, E11.641, E11.649, E13.641, E13.649, E15, E160, E161, E162, T383X1A, T383X1D, T383X1S, T383X2A, T383X2D, T383X2S, T383X3A, T383X3D, T383X3S, T383X4A, T383X4D, T383X4S, T383X5A, T383X5D, T383X5S
**DKA/HHS**	250.1x, 250.2x	E10.10, E10.11, E11.00, E11.01, E11.641, E13.10

Abbreviations: DKA, diabetic ketoacidosis; HHS, hyperglycemic hyperosmolar state.

© 2022 Kurani SS et al. *JAMA Network Open.*

eTable 3. Classification of Glucose-Lowering Medications

Combination medications were considered as separate agents belonging to each ingredient class.

Medication class	Included agents
**Biguanides**	Metformin
**Sulfonylureas**	Glimepiride<br>Glipizide<br>Glyburide
**Thiazolidinediones**	Pioglitazone<br>Rosiglitazone
**DPP-4 inhibitors**	Alogliptin<br>Linagliptin<br>Sitagliptin<br>Saxagliptin
**GLP-1 receptor analogs**	Exenatide<br>Liraglutide<br>Albiglutide<br>Dulaglutide<br>Semaglutide<br>Lixisenatide
**SGLT2 inhibitors**	Canagliflozin<br>Empagliflozin<br>Dapagliflozin<br>Ertugliflozin
**Glinides**	Nateglinide<br>Repaglinide
**Amylin analogs**	Pramlintide
**α-Glucosidase inhibitors**	Acarbose<br>Miglitol
**Basal insulin**	<u>Human</u><br>NPH<br>Isophane<br><br><u>Analog</u><br>Detemir<br>Glargine<br>Degludec
**Bolus insulin**	<u>Human</u><br>Regular<br><br><u>Analog</u><br>Aspart<br>Lispro<br>Glulisine<br>Inhaled powder insulin

© 2022 Kurani SS et al. *JAMA Network Open.*

eTable 4. Risk Factors of Severe Hypoglycemia and DKA/HHS (Sensitivity Analysis Using the Partially Adjusted Model)

Results of negative binomial regression analyses adjusted only for patient demographics (age, sex, health plan) and percent White residents in the county.

	Hypoglycemia		DKA/HHS	
	**IRR (95% CI)**	**p-value**	**IRR (95% CI)**	**p-value**
**Percent White residents in county**	0.64 (0.51-0.82)	<0.001	0.78 (0.62-0.99)	0.04
**ADI quintile**				
Q1 (least deprived)	Ref		Ref	
Q2	1.00 (0.91-1.09)	0.96	1.02 (0.92-1.12)	0.75
Q3	1.17 (1.06-1.28)	0.002	1.24 (1.11-1.37)	<0.001
Q4	1.15 (1.03-1.29)	0.02	1.12 (0.99-1.26)	0.07
Q5 (most deprived)	1.48 (1.33-1.64)	<0.001	1.11 (0.99-1.26)	0.08
**Sex**				
Female	Ref		Ref	
Male	0.93 (0.90-0.96)	<0.001	0.84 (0.79-0.88)	<0.001
**Health Plan**				
Commercial	Ref		Ref	
Medicare Advantage	4.82 (4.45-5.21)	<0.001	3.34 (3.05-3.65)	<0.001
**Age, years**				
18-44	Ref		Ref	
45-64	0.58 (0.53-0.64)	<0.001	0.17 (0.15-0.18)	<0.001
65-74	0.35 (0.32-0.39)	<0.001	0.04 (0.04-0.05)	<0.001
≥75	0.49 (0.44-0.54)	<0.001	0.04 (0.03-0.04)	<0.001

Abbreviations: ADI, area deprivation index; DKA, diabetic ketoacidosis; HHS, hyperglycemic hyperosmolar state.

© 2022 Kurani SS et al. *JAMA Network Open.*

eTable 5. Estimated Rates of Severe Hypoglycemia and DKA/HHS (Sensitivity Analysis Using the Partially Adjusted Model)

Rates of severe hypoglycemia and DKA/HHS per 1000 person-years, analyses adjusted only for patient demographics (age, sex, health plan) and percent White residents in the county.

	Hypoglycemia<br>Rate per 1000 PY (95% CI)	DKA/HHS<br>Rate per 1000 PY (95% CI)
**ADI quintile**		
Q1 (least deprived)	11.16 (10.60-11.73)	5.73 (5.38-6.09)
Q2	11.14 (10.22-12.05)	5.83 (5.34-6.32)
Q3	13.01 (11.91-14.10)	7.08 (6.45-7.71)
Q4	12.86 (11.53-14.19)	6.40 (5.73-7.07)
Q5 (most deprived)	16.49 (15.09-17.89)	6.39 (5.72-7.05)

Abbreviations: ADI, area deprivation index; DKA, diabetic ketoacidosis; HHS, hyperglycemic hyperosmolar state; PY, person-years.

© 2022 Kurani SS et al. *JAMA Network Open.*

eTable 6. Estimated Rates of Severe Hypoglycemia and DKA/HHS in Subgroups of Patient Age

Rates of severe hypoglycemia and DKA/HHS per 1000 person-years, analyses adjusted for patient demographics (age, sex, health plan), percent White residents in the county, comorbidities, and medications (fully adjusted model). *p <0.05 for the IRR of the quintile examined (Q2-Q5) with ADI Q1 (least deprived) serving as the reference group.

	Hypoglycemia		DKA/HHS	
	**IRR (95% CI)**	**Rate per 1000 PY (95% CI)**	**IRR (95% CI)**	**Rate per 1000 PY (95% CI)**
**18-44 years old**				
Q1 (least deprived)	Ref	11.42 (9.45-13.39)	Ref	28.93 (25.28-32.57)
Q2	1.16 (0.97-1.38)	13.21 (10.75-15.66)	1.09 (0.92-1.29)	31.51 (26.80-36.21)
Q3	0.90 (0.73-1.10)	10.26 (8.16-12.37)	1.17 (0.99-1.39)	33.87 (28.98-38.75)
Q4	1.27 (1.03-1.57)*	14.54 (11.64-17.44)	1.31 (1.07-1.60)*	37.80 (30.97-44.64)
Q5 (most deprived)	1.24 (0.94-1.65)	14.22 (10.59-17.85)	1.42 (1.11-1.82)*	40.99 (31.26-50.72)
**45-64 years old**				
Q1 (least deprived)	Ref	11.61 (10.66-12.56)	Ref	7.75 (6.88-8.61)
Q2	0.96 (0.87-1.07)	11.17 (10.08-12.27)	1.04 (0.93-1.17)	8.08 (7.20-8.97)
Q3	1.05 (0.94-1.17)	12.16 (10.87-13.45)	1.15 (1.01-1.30)*	8.88 (7.76-9.99)
Q4	1.03 (0.91-1.17)	11.99 (10.54-13.44)	1.06 (0.92-1.21)	8.18 (7.14-9.21)
Q5 (most deprived)	1.29 (1.14-1.46)*	15.00 (13.24-16.76)	1.00 (0.84-1.19)	7.76 (6.47-9.05)
**65-74 years old**				
Q1 (least deprived)	Ref	13.42 (12.55-14.30)	Ref	4.11 (3.49-4.74)
Q2	0.97 (0.88-1.07)	13.03 (11.85-14.21)	0.94 (0.81-1.10)	3.88 (3.33-4.43)
Q3	1.14 (1.02-1.27)*	15.33 (13.90-16.75)	1.12 (0.95-1.33)	4.62 (3.91-5.32)
Q4	1.20 (1.05-1.37)*	16.05 (14.04-18.05)	1.14 (0.95-1.35)	4.67 (3.90-5.44)
Q5 (most deprived)	1.37 (1.22-1.54)*	18.44 (16.64-20.23)	1.10 (0.92-1.33)	4.53 (3.79-5.28)
**≥75 years old**				
Q1 (least deprived)	Ref	17.79 (16.71-18.87)	Ref	3.18 (2.83-3.53)
Q2	1.06 (0.96-1.16)	18.77 (17.19-20.35)	0.99 (0.86-1.15)	3.15 (2.73-3.58)
Q3	1.18 (1.07-1.31)*	21.05 (19.23-22.88)	1.09 (0.95-1.26)	3.47 (3.03-3.91)
Q4	1.17 (1.03-1.32)*	20.75 (18.47-23.03)	1.09 (0.91-1.31)	3.47 (2.86-4.08)
Q5 (most deprived)	1.55 (1.38-1.74)*	27.60 (24.82-30.39)	1.13 (0.95-1.35)	3.59 (3.01-4.17)

Abbreviations: ADI, area deprivation index; DKA, diabetic ketoacidosis; HHS, hyperglycemic hyperosmolar state; PY, person-years.

© 2022 Kurani SS et al. *JAMA Network Open.*

eTable 7. Estimated Rates of Severe Hypoglycemia and DKA/HHS in Subgroups of Patient Sex

Rates of severe hypoglycemia and DKA/HHS per 1000 person-years, analyses adjusted for patient demographics (age, sex, health plan), percent White residents in the county, comorbidities, and medications (fully adjusted model). *p <0.05 for the IRR of the quintile examined (Q2-Q5) with ADI Q1 (least deprived) serving as the reference group.

	**Hypoglycemia**		**DKA/HHS**	
	**IRR (95% CI)**	**Rate per 1000 PY (95% CI)**	**IRR (95% CI)**	**Rate per 1000 PY (95% CI)**
**Men**				
Q1 (least deprived)	Ref	12.52 (11.88-13.15)	Ref	7.22 (6.42-8.01)
Q2	1.00 (0.93-1.08)	12.51 (11.64-13.37)	0.95 (0.85-1.07)	6.87 (6.06-7.68)
Q3	1.11 (1.01-1.22)*	13.89 (12.65-15.12)	1.08 (0.96-1.21)	7.80 (6.82-8.77)
Q4	1.11 (1.01-1.23)*	13.95 (12.66-15.25)	1.05 (0.93-1.20)	7.60 (6.66-8.55)
Q5 (most deprived)	1.32 (1.19-1.47)*	16.57 (14.98-18.16)	1.06 (0.91-1.23)	7.63 (6.55-8.72)
**Women**				
Q1 (least deprived)	Ref	14.54 (13.63-15.46)	Ref	7.75 (7.09-8.42)
Q2	1.01 (0.92-1.11)	14.74 (13.63-15.85)	1.09 (0.99-1.20)	8.46 (7.71-9.21)
Q3	1.13 (1.02-1.25)*	16.48 (15.03-17.93)	1.19 (1.07-1.32)*	9.20 (8.27-10.13)
Q4	1.18 (1.06-1.32)*	17.21 (15.45-18.96)	1.19 (1.06-1.35)*	9.25 (8.19-10.31)
Q5 (most deprived)	1.48 (1.34-1.63)*	21.51 (19.72-23.31)	1.17 (1.02-1.35)*	9.09 (7.86-10.31)

Abbreviations: ADI, area deprivation index; DKA, diabetic ketoacidosis; HHS, hyperglycemic hyperosmolar state; PY, person-years.

© 2022 Kurani SS et al. *JAMA Network Open.*

eTable 8. Estimated Rates of Severe Hypoglycemia and DKA/HHS in Subgroups of Glucose-Lowering Therapy

Rates of severe hypoglycemia and DKA/HHS per 1000 person-years, analyses adjusted for patient demographics (age, sex, health plan), percent White residents in the county, comorbidities, and medications (fully adjusted model). *p <0.05 for the IRR of the quintile examined (Q2-Q5) with ADI Q1 (least deprived) serving as the reference group.

	Hypoglycemia		DKA/HHS	
	**IRR (95% CI)**	**Rate per 1000 PY (95% CI)**	**IRR (95% CI)**	**Rate per 1000 PY (95% CI)**
**Bolus ± basal insulin**				
Q1 (least deprived)	Ref	43.04 (40.79-45.29)	Ref	25.97 (23.87-28.07)
Q2	1.01 (0.94-1.10)	43.68 (40.69-46.66)	1.11 (0.99-1.24)	28.82 (26.02-31.62)
Q3	1.03 (0.95-1.12)	44.44 (41.27-47.62)	1.25 (1.13-1.39)*	32.52 (29.84-35.19)
Q4	1.10 (1.00-1.21)	47.19 (43.17-51.2)	1.16 (0.99-1.35)	30.11 (26.01-34.21)
Q5 (most deprived)	1.30 (1.18-1.43)*	55.79 (51.02-60.57)	1.16 (0.98-1.38)	30.20 (25.64-34.77)
**Basal insulin**				
Q1 (least deprived)	Ref	22.89 (21.26-24.52)	Ref	9.40 (8.17-10.64)
Q2	1.01 (0.91-1.12)	23.14 (21.39-24.89)	0.93 (0.78-1.13)	8.78 (7.35-10.21)
Q3	1.19 (1.06-1.33)*	27.14 (24.52-29.77)	1.04 (0.88-1.24)	9.81 (8.28-11.34)
Q4	1.18 (1.04-1.34)*	26.96 (24.06-29.86)	1.19 (0.99-1.42)	11.15 (9.43-12.87)
Q5 (most deprived)	1.33 (1.18-1.50)*	30.45 (27.33-33.57)	1.16 (0.94-1.44)	10.91 (8.76-13.07)
**Non-insulin drugs only**				
Q1 (least deprived)	Ref	5.44 (5.11-5.77)	Ref	1.67 (1.51-1.83)
Q2	1.03 (0.93-1.15)	5.63 (5.14-6.11)	0.99 (0.87-1.14)	1.66 (1.49-1.83)
Q3	1.11 (0.99-1.24)	6.03 (5.44-6.62)	1.07 (0.93-1.23)	1.79 (1.61-1.96)
Q4	1.13 (1.00-1.28)	6.15 (5.44-6.85)	1.07 (0.93-1.25)	1.79 (1.58-2.01)
Q5 (most deprived)	1.48 (1.32-1.65)*	8.03 (7.29-8.77)	1.01 (0.86-1.20)	1.69 (1.47-1.92)

Abbreviations: ADI, area deprivation index; DKA, diabetic ketoacidosis; HHS, hyperglycemic hyperosmolar state; PY, person-years.

© 2022 Kurani SS et al. *JAMA Network Open.*

eTable 9. Estimated Rates of Severe Hypoglycemia and DKA/HHS in Subgroups of Patient Health Plans

Rates of severe hypoglycemia and DKA/HHS per 1000 person-years, analyses adjusted for patient demographics (age, sex, health plan), percent White residents in the county, comorbidities, and medications (fully adjusted model). *p <0.05 for the IRR of the quintile examined (Q2-Q5) with ADI Q1 (least deprived) serving as the reference group.

	Hypoglycemia		DKA/HHS	
	**IRR (95% CI)**	**Rate per 1000 PY (95% CI)**	**IRR (95% CI)**	**Rate per 1000 PY (95% CI)**
**Commercial insurance**				
Q1 (least deprived)	Ref	6.02 (5.51-6.52)	Ref	8.01 (7.31-8.72)
Q2	0.97 (0.86-1.10)	5.85 (5.18-6.53)	1.10 (0.98-1.25)	8.86 (7.84-9.87)
Q3	0.97 (0.86-1.11)	5.87 (5.12-6.61)	1.16 (1.05-1.30)*	9.33 (8.42-10.24)
Q4	0.99 (0.83-1.19)	5.98 (4.89-7.06)	1.15 (0.96-1.36)	9.18 (7.72-10.64)
Q5 (most deprived)	1.31 (1.10-1.56)*	7.89 (6.51-9.26)	1.25 (1.05-1.50)*	10.04 (8.33-11.74)
**Medicare Advantage**				
Q1 (least deprived)	Ref	17.73 (16.8-18.67)	Ref	7.02 (6.30-7.74)
Q2	1.02 (0.94-1.11)	18.03 (16.75-19.31)	0.96 (0.86-1.07)	6.72 (6.00-7.44)
Q3	1.16 (1.05-1.27)*	20.52 (18.87-22.17)	1.12 (1.00-1.24)	7.83 (6.95-8.70)
Q4	1.18 (1.07-1.31)*	20.99 (19.03-22.95)	1.11 (0.98-1.26)	7.80 (6.86-8.74)
Q5 (most deprived)	1.43 (1.30-1.57)*	25.3 (23.27-27.32)	1.07 (0.93-1.22)	7.49 (6.53-8.46)

Abbreviations: ADI, area deprivation index; DKA, diabetic ketoacidosis; HHS, hyperglycemic hyperosmolar state; PY, person-years.

© 2022 Kurani SS et al. *JAMA Network Open.*

eReference

1. Ginde AA, Blanc PG, Lieberman RM, Camargo CA, Jr. Validation of ICD-9-CM coding algorithm for improved identification of hypoglycemia visits. BMC Endocr Disord 2008;8:4

© 2022 Kurani SS et al. *JAMA Network Open.*
